# The GC-content at the 5′ ends of human protein-coding genes is undergoing mutational decay

**DOI:** 10.1186/s13059-024-03364-x

**Published:** 2024-08-13

**Authors:** Yi Qiu, Yoon Mo Kang, Christopher Korfmann, Fanny Pouyet, Andrew Eckford, Alexander F. Palazzo

**Affiliations:** 1 Department of Biochemistry, University of Toronto, Toronto, Ontario M5G1M1 Canada; 2 Department of Electrical Engineering and Computer Science, York University, Toronto, Ontario M3J1P3 Canada; 3 Laboratoire Interdisciplinaire des Sciences du Numérique, Université Paris-Saclay, 91190 Gif-sur-Yvette, France

**Keywords:** GC-content, Genome evolution, Mutational bias, PRDM9, GC-biased gene conversion, Recombination

## Abstract

**Background:**

In vertebrates, most protein-coding genes have a peak of GC-content near their 5′ transcriptional start site (TSS). This feature promotes both the efficient nuclear export and translation of mRNAs. Despite the importance of GC-content for RNA metabolism, its general features, origin, and maintenance remain mysterious. We investigate the evolutionary forces shaping GC-content at the transcriptional start site (TSS) of genes through both comparative genomic analysis of nucleotide substitution rates between different species and by examining human de novo mutations.

**Results:**

Our data suggests that GC-peaks at TSSs were present in the last common ancestor of amniotes, and likely that of vertebrates. We observe that in apes and rodents, where recombination is directed away from TSSs by PRDM9, GC-content at the 5′ end of protein-coding gene is currently undergoing mutational decay. In canids, which lack PRDM9 and perform recombination at TSSs, GC-content at the 5′ end of protein-coding is increasing. We show that these patterns extend into the 5′ end of the open reading frame, thus impacting synonymous codon position choices.

**Conclusions:**

Our results indicate that the dynamics of this GC-peak in amniotes is largely shaped by historic patterns of recombination. Since decay of GC-content towards the mutation rate equilibrium is the default state for non-functional DNA, the observed decrease in GC-content at TSSs in apes and rodents indicates that the GC-peak is not being maintained by selection on most protein-coding genes in those species.

**Supplementary Information:**

The online version contains supplementary material available at 10.1186/s13059-024-03364-x.

## Background

One of the most striking features of human protein-coding genes is the pattern of GC-content present along its length [1, 2]. In particular, it has been observed that GC-content is highest at the 5′ end of genes, and that this decreases as one travels downstream and is lowest at the 3′ end of genes [1–8]. In addition, it has been observed that GC-content is higher in exons than in introns, with it being highest in the first exon and decreasing with every subsequent exon [1, 8]. This is also true in introns, with GC-content highest in the first, and decreasing with every subsequent intron [1, 8].

GC-content appears to play several different roles in the gene expression pathway. Clusters of CpG dinucleotides, called CpG islands, are found near the TSS and are associated with robust and high expression level of genes, including genes expressed in many tissues, such as housekeeping genes [9]. CpG-rich promoters have been shown to activate transcription by recruiting specific transcription factors [10]. Moreover, differences between GC-content in exons and introns enhance splicing [11]. Finally, high GC-content, when present at the 5′ end of intron-poor mRNAs, promotes mRNA nuclear export [2, 12, 13]. Indeed, it has been noted that RNA elements that are GC-rich tend to promote nuclear export when inserted at the 5′ ends of intronless reporter mRNAs [14–17]. These GC-rich regions likely recruit protein factors, such as SARNP, SR proteins, and RBM33, which directly recruit nuclear transport receptors, like NXF1/NXT1, that ferry the mRNAs across the nuclear pore [18–21]. This GC-dependent nuclear export is likely also important for the nuclear export of certain long non-coding RNAs, such as *NORAD*, which are produced from intronless genes [20, 22]. In contrast, mRNAs from intron-rich genes largely acquire nuclear export factors during splicing [22, 23]. As a result, highly spliced mRNAs are exported in a GC-content-independent manner [13].

The forces that dictate GC-content in genes remain unclear. There is some evidence suggesting that GC-content is partially shaped by adaptive evolutionary forces. This comes from the study of de novo genes that are generated when mRNAs are inadvertently copied to cDNA and then reinserted into the genome to form active “retrogenes” [13]. Indeed, it has been observed that these genes have elevated GC-content at their 5′ ends in comparison to their intron-containing counterparts, suggesting that an increase in GC-content can be driven by positive selection to drive their efficient nuclear export [13]. Moreover, retrogenes tend to arise from parental genes that have high GC-content at their 5′ ends suggesting that this feature may help promote their expression and make it less likely that they convert to pseudogenes [24]. This trend, however, seems restricted to the gene body as retrogene promoters tend to have fewer CpGs than parental genes [25]. Other observations suggest that GC-content at the start of genes is influenced by non-adaptive forces. First, highly spliced mRNAs tend to have high GC-content at their 5′ ends despite the fact that GC-rich regions are not required for their nuclear export and appear not to affect how much protein they produce [13]. Second, GC-content extends for quite some distance upstream from the transcriptional start site beyond the promoter region [4, 5] and downstream into the first intron [1, 8], which are under little selection. Thus, it is likely that GC-content is also shaped by non-adaptive forces.

One of these non-adaptive forces is the regional differences in mutation rates along genes. For example, it has been shown that CpG dinucleotides surrounding the TSS of active human genes experience fewer substitutions [26], likely due to the fact that they are hypomethylated, and that this protects them from mutational decay due to the enhanced repair of deaminated cytosines compared to 5-methylated cytosines [27]. This decrease in CpG decay may also be due to higher melting temperatures in GC-rich regions which lowers the propensity that DNA is single stranded and susceptible to cytosine deamination [28]. As CpGs only account for a small fraction of all GC-content surrounding the TSS, even in CpG-rich promoters, this form of mutational bias does not fully explain the pattern of GC-content seen at the beginning of most human genes.

Another major non-adaptive evolutionary force that can influence local GC-content is GC-biased gene conversion (gBGC) [29]. This process occurs due to the formation of heteroduplex regions between maternal and paternal chromosomes during homologous recombination. When the two chromosomes are heterozygous, mismatches will form in the heteroduplex which are typically corrected in favor of Gs and Cs over As and Ts by about 70% in humans [30, 31]. As a result, G and C single nucleotide polymorphisms surrounding recombination sites are more likely to be transmitted to the next generation and spread more rapidly in the population thus resembling positive selection. Importantly, recombination is concentrated at certain “hotspots” which contain nucleotide motifs that are bound by PRDM9, a histone methyltransferase [32, 33]. PRDM9 in turn recruits the topoisomerase SPO11 to generate double stranded breaks that initiate homologous recombination [34]. In this way, PRDM9 binding dictates where recombination occurs, which in turn elevates local gBGC, that leads to an increase in GC-content [29].

There are interesting connections between recombination hotspots and TSSs. PRDM9 promotes the trimethylation of H3K4 and H3K36, with the former but not the later, also being present at TSSs. Deletion of PRDM9 in mice led to a shift in recombination from hotspots to TSSs [35, 36]. And finally, although PRDM9 is found throughout the eukaryotic tree, some lineages, such as canids and birds, have lost this gene and perform recombination at TSSs [37, 38].

PRDM9 and recombination hotspots experience accelerated rates of evolution due to the rapid elimination of PRDM9 motifs. This is driven by either double stranded break-driven biased gene conversion (dBGC) [39, 40], or other processes [41]. Subsequently there is selective pressure on PRDM9 to recognize new motifs [33]. Thus, the effects of recombination, including gBGC, at any one particular loci are short-lived in evolutionary time, but can affect large parts of the genome over extended times as the concentration of recombination events shifts from one loci to the next.

Here we characterize the GC-content surrounding the TSS of protein-coding genes and infer the nucleotide substitution dynamics of these regions. Although high GC-content in certain protein-coding genes may be shaped in part by selective pressures to promote mRNA nuclear export and translation, our data suggests that this feature is currently under decay in humans, chimpanzees, and rodents, while it is growing in canids. Decay of GC-content towards equilibrium is the default state for non-functional DNA that is GC-rich. That this decay is occurring surrounding the TSS in some mammals is surprising as it indicates that the GC-peak is not being maintained by selection in those species. Our results, along with other recently published findings, indicate that the GC-peak present at the TSS in amniotes, and likely most vertebrates, is largely influenced by historic patterns of recombination.

## Results

### Analysis of the GC-peak in human protein-coding genes

**Fig. 1:**
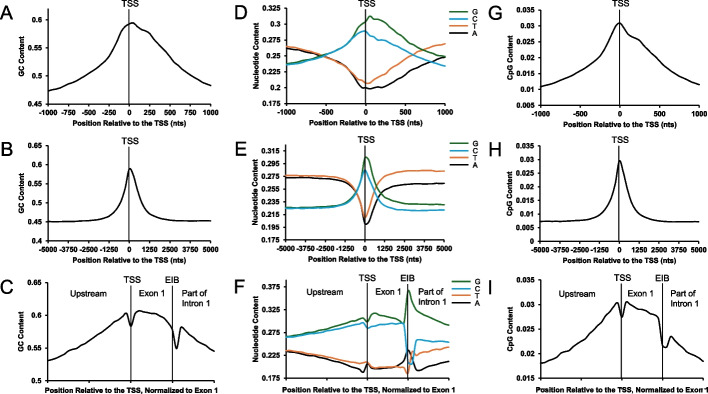
Nucleotide content of genomic regions surrounding TSS sites of human protein-coding genes. For all annotated human protein-coding genes (*N* = 18,874), the average GC-content (**A**–**C**), nucleotide content on the coding strand (**D**–**F**), and CpG-content (**G**–**I**) were plotted (*y-axis*) against the nucleotide position surrounding the TSS (*x-axis*; 2 kb surrounding the TSS in **A**, **D**, and **G**; 10 kb surrounding the TSS in **B**, **E**, and **H**) with negative numbers indicating upstream sequence and positive numbers indicating downstream sequence, or along binned genomic sequence the TSSs and first exon-intron boundaries (EIB) of all human protein-coding genes were aligned so that each gene sequence was “normalized” for the length of the first exon (*x-axis* **C**, **F**, and **I**)

It is known that in human protein-coding genes, GC-content peaks around the transcription start site (TSS) and slopes down into both the upstream intergenic region and downstream into the first exon and even the first intron [1–8]. We wanted to examine this GC-peak more closely by metagene analysis and compare it to other gene landmarks. As reported elsewhere, GC-content is highest just downstream of the TSS and descends more-or-less symmetrically on both sides until it plateaus off at 45% (Fig. 1A, B). Note that the genome average GC-content is roughly 41%, but that genes tend to be enriched in GC-rich isochores (genomic regions elevated in GC-content) [42].

To get a sense of how GC-content varied with respect to the nearby landmarks, we repeated the analysis of human protein-coding genes but this time we normalized each region to the length of the first exon. We also analyzed an equal amount of sequence into the first intron to examine the GC-content surrounding the exon-intron boundary (EIB). In addition, we extended the analysis upstream from the TSS to examine GC-content at the intergenic-exon boundary. Again, we observed that GC-content peaks within the first exon (Fig. 1C). Notably, the GC-peak forms a nearly normal curve where it slopes down into both the upstream intergenic region and downstream into the first intron with near identical slopes. This curve is interrupted by the TSS and the exon-intron boundary which are marked by features that are both depressed in GC-content. The dip at the TSS is likely due to the fact that transcription of most genes begins with purines [43, 44] and this slightly elevates the number of adenines in this region. The dip associated with the exon-intron boundary is likely due to the presence of the 5′ splice site motif, which is slightly GC-poor [45].

Next, we examined the content of all four nucleotides over these regions. In particular, we wanted to assess any strand asymmetry which manifests as a difference between cognate nucleotides (for example, G and C) in each strand. As reported elsewhere, cognate nucleotides were mostly similar upstream of the TSS (Fig. 1D) and leveled off at long distances (>2.5 kbps) (Fig. 1E). Interestingly, divergence of cognate nucleotides was apparent just upstream of the TSS (within 120 bps) and this divergence increased downstream of the TSS until it reached a stable level after long distances (>2.5 kbps) (Fig. 1D, E). Since the average exon is only ~200 bps while introns are about an order of magnitude longer, most of the observed strand asymmetries >200 bps after the TSS are due to the nucleotide content of the introns. Because these are not under strong selection pressures, the observed asymmetry must be due to strong strand-specific mutation/repair biases that counters mutational decay, which would normally reverse these asymmetries. These biases include transcription-induced damage and/or transcription-coupled DNA repair of the template strand, and enhanced chemical modification of the coding strand, which is single stranded during transcription. Similar arguments have been advanced by other groups [26].

When nucleotide composition was examined with respect to the first exon and its delimiting landmarks, further patterns could be discerned (Fig. 1F). Right at the TSS, A-content slightly increases likely due to the need for a purine at the first position of the transcript. Within the first exon, A and T displayed little strand asymmetry while G and C diverge—this asymmetry peaks just after the transcriptional start site and then diminishes towards the end of the exon. At the exon-intron boundary, the content of all four nucleotides drastically changes, likely due to the presence of the 5′ splice site motif. Finally, within the intron, strand asymmetries start off very high and then trend towards the strand asymmetries seen in the body of the intron (as seen in Fig. 1E).

Next, we examined the level of CpG dinucleotides whose cytosines are often methylated to form 5-methylcytosine. When methylated, CpG are prone to mutational decay as the spontaneous deamination of 5-methylcytosine forms T [46]. Importantly, CpGs tend to be demethylated around the TSS of active genes, thus reducing their mutational decay. As described previously [26], CpG dinucleotides are enriched surrounding the TSS and generally mirror the overall GC-content (Fig. 1G–I). Note that CpG dinucleotides make up at most 3% of all sequences around the TSS and thus cannot fully explain the presence of a GC-peak.

### GC-peaks at the 5′ end of genes are present in most vertebrates

**Fig. 2:**
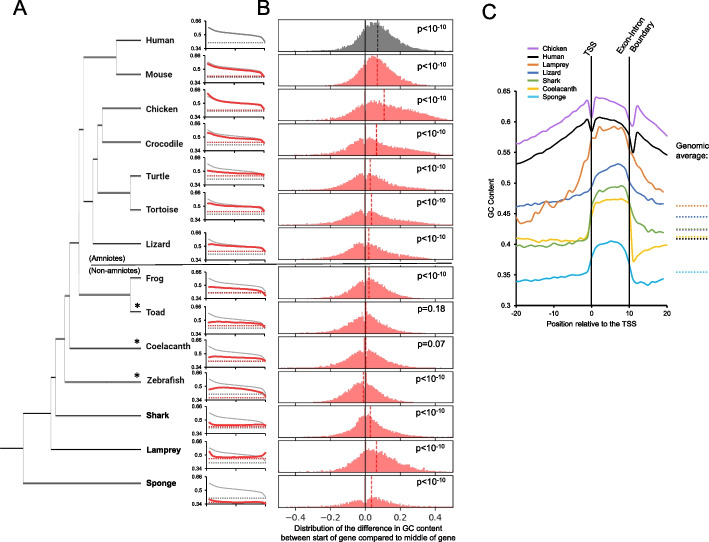
Analysis of GC-content of mRNAs and TSSs from various vertebrate genomes. **A** For all annotated mRNAs of the indicated species (red solid lines), the average GC-content (*y-axis*) was plotted against the normalized mRNA length (from 5′ end to 3′ end in 20 bins; *x-axis*). Genome average GC-content was also plotted (red dashed lines). As a comparison, GC-content for human mRNA (gray solid lines) and human genome (gray dashed lines) were replotted in each graph. Phylogenetic relationships of the organisms are indicated on the left. **B** For each indicated species, the bar graph displays the fraction of mRNAs (*y-axis*) with various differences in GC-content between the start (bin 1) and the middle (bin 11) of the mRNA (total bins 20; *x-axis*). Average difference in GC-content for all mRNAs in a given species is indicated by the dashed line. Wilcoxon signed-rank test was performed for the GC-content at the start of the mRNA and the middle of the mRNA, and *p* values are indicated for each species analyzed. **C** For all annotated protein-coding genes of the indicated species, a metaplot of the average GC-content (*y-axis*) along genomic sequence, normalized in length to align all TSSs and first exon-intron boundaries (as described in Fig. 1C; *x-axis*). As a comparison, genome average GC-content was also plotted (dashed lines on the right)

To learn how the GC-content of mRNAs varies across vertebrates, we performed a metagene analysis of GC-content along the length of mRNAs from all protein-coding genes from several genomes and compared this to human mRNAs (Fig. 2A, for comparison human plots are in gray, species-specific plots are in red). In all vertebrates, GC-content in mRNAs was much higher than the genome average (Fig. 2A, dotted lines). We calculated the differences between GC-content at the beginning of the mRNA compared to the middle of the mRNA for all genes analyzed, and tested whether the distribution of these differences is significantly higher than 0 (Fig. 2B). We observed that in amniotes, GC-content was significantly higher at the 5′ end of the mRNA, and the GC-levels gradually decreased downstream (Fig. 2A, B). We also observed that this GC-peak was missing in several non-amniotes, including toads, coelacanth, and zebrafish (Fig. 2A, B). Since it is present in non-boney fish (e.g., shark and lamprey), it is possible that the GC-peak was present in the vertebrate common ancestor and lost several times (e.g., toads, coelacanths, and zebrafish, see asterisks). Of course, our ability to detect these trends relies heavily on the correct annotation of these genomes as the majority of the GC-peak should be within the 5′ UTR. Nevertheless, we can still detect the GC-peak in human mRNAs if we restrict ourselves to the open reading frames (ORFs; Additional file 1, Fig. S1), and this remains true even if we parse out mRNAs that encode a signal sequence, as the signal sequence coding region is known to be GC-rich and found at the 5′ end of the ORF [14, 47, 48]. All species, with the possible exception of turtles and lamprey, had a dip in GC-content at the 3′ end of their mRNAs (Fig. 2A).

We next examined GC-content surrounding the first exon and its associated landmarks to examine the GC-peak more closely in a select number of species (Fig. 2C). For both humans and chicken, GC-content peaks within the first exon and forms a nearly normal curve where it slopes down into both the upstream intergenic region and downstream into the first intron. Despite this, GC-content in exons, introns, and upstream intergenic regions were much higher than the genome average (see dashed lines). Interestingly, this pattern is different in other vertebrates, including anole lizards, coelacanth, shark, and lamprey. These organisms had clear differences in GC-content between their first exon and surrounding sequences (upstream and intronic sequences), which came close to the overall genomic GC-content. It is thus likely that a subset of amniotes experienced specific evolutionary forces that elevated GC-content in regions surrounding the TSS (i.e., in introns and upstream intergenic regions), although we cannot rule out the possibility that these trends are more ancestral to amniotes and were lost in certain lineages and hard to see in others. Indeed, if this trend was present in the amniote ancestor, it has been mostly lost in anole lizards.

### GC-content in genes correlates with recombination

**Fig. 3:**
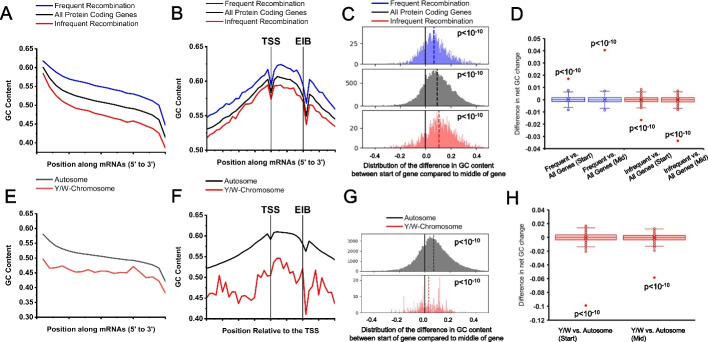
Effects of recombination on the GC-content of mRNAs and TSSs. **A** For each set of annotated human mRNAs, the average GC-content (*y-axis*) was plotted against the normalized mRNA length (from 5′ end to 3′ end in 20 bins; *x-axis*). mRNA transcribed from human genes with the 10% highest (blue line) and 10% lowest (red line) recombination frequencies were plotted. The average of all mRNAs was also plotted (black line). **B** For each set of annotated human protein-coding genes described in **A**, the average GC-content (*y-axis*) was plotted against genomic sequence normalized to the TSS and first exon-intron boundary (EIB) over 41 bins (as described in Fig. 1C; *x-axis*). **C** Wilcoxon signed-rank test was performed for the GC-content at the start of the mRNA and the middle of the mRNA described in **A**, and *p* values were indicated. **D** Permutation tests between either the start of the mRNA in test groups described in **A**, or middle of the mRNA in test groups described in **A**. Distribution of differences from 1000 randomized permutations are displayed in box and whisker plots. Actual differences are plotted with red dots. **E** For each set of annotated mRNAs, the average GC-content (*y-axis*) was plotted against the normalized mRNA length of (from 5′ end to 3′ end in 20 bins; *x-axis*). mRNAs transcribed from the autosomes of 5 species (human, mouse, rat, pig, chicken; black line) were compared to mRNAs transcribed from the Y (human, mouse, rat, pig) or W (chicken) chromosomes. **F** For each set of annotated protein-coding genes described in **E**, the average GC-content (*y-axis*) was plotted against genomic sequence normalized to the TSS and first exon-intron boundary (EIB) over 41 bins (as described in Fig. 1C; *x-axis*). **G** Wilcoxon signed-rank test was performed for the GC-content at the start of the mRNA and the middle of the mRNA described in **E**, and *p* values were indicated. **H** Permutation tests between either the start of the mRNA in test groups described in **E**, or middle of the mRNA in test groups described in **E**. Distribution of differences from 1000 randomized permutations are displayed in box and whisker plots. Actual differences are plotted with red dots

Recombination is known to elevate GC-content through gBGC [29]. Although gBGC is thought to be widespread in animals [49], it has specific effects that appear to be amniote-specific [50]. To examine whether the frequency of recombination correlates with the GC-peak, we analyzed the GC-content of mRNAs from human genes divided into two categories: those that undergo frequent recombination (top 10%) and those that undergo infrequent recombination (bottom 10%) [51]. The GC-content of mRNAs undergoing frequent recombination is significantly higher than the average, all along the length of the mRNA (Fig. 3A), including at the 5′ end (Fig. 3A, B, D) and at the midpoint (Fig. 3A, D). The opposite is true for mRNAs undergoing infrequent recombination (Fig. 3A, B, D). The same trend is observed when examining these genes around their TSS; however, all genes, whether or not recombination is frequent, have a GC-peak at the 5′ end that is significantly higher in GC-content than the middle of the gene (Fig. 3C). These results suggest that recombination affects GC-content throughout the entire length of the gene and not just at the TSS. However, since genes that undergo the least amount of recombination have the highest relative GC-peak (GC-content at the 5′ end compared to the middle of the gene), it is unlikely that current recombination patterns are responsible for the presence of the peak.

To further explore this relationship, we next examined protein-coding mRNAs from the Y chromosome, which is not subjected to recombination events. Since there are few genes on the human Y, we compiled genes from Y (mammals) and W (for birds) chromosomes from five species (humans, mice, rat, pigs, and chickens). Similar to mRNAs undergoing infrequent recombination, GC-content for Y/W chromosome mRNAs is significantly lower around the TSS and throughout their entire length when compared to those from autosome genes (Fig. 3E–H). There is still a significant GC-peak in the Y/W chromosome genes (Fig. 3G); however, the GC-peak at the 5′ of the mRNAs is less drastic in Y/W chromosomes compared to autosomes (Fig. 3G, H).

Overall, the data are in line with the idea that recombination can affect GC-content, although it does not appear that this explains the GC-peak.

### *Primates* and rodents are experiencing a loss of GCs surrounding protein-coding gene TSSs

**Fig. 4:**
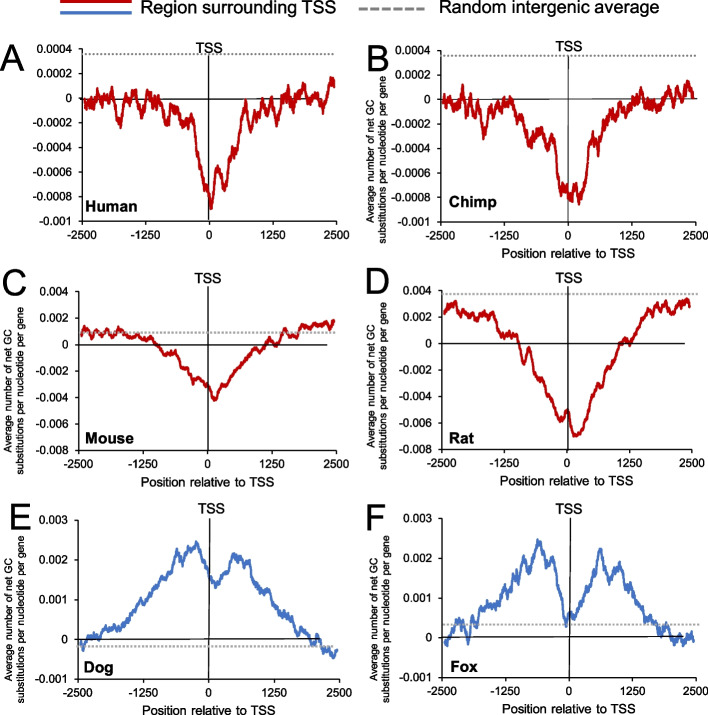
Changes in GC-content surrounding TSSs from various mammalian genomes according to comparative phylogenetic analysis. **A**–**B** Substitution events were inferred by aligning sequences surrounding the TSS of 9248 human and chimpanzee protein-coding genes, using the gorilla sequence to infer the ancestral state. The total change in Gs and Cs in human (**A**) and chimpanzee (**B**) were compiled and divided by the number of genes analyzed (*y-axis*) using a sliding window of 100 bp and plotted along genomic regions surrounding the TSS (*x-axis*). The overall average changes in GC-content in random intergenic (dotted line—see Additional file 1, Fig. S3A–B) were plotted. **C**–**D** Similar to **A**–**B** except that 4840 mouse and rat genes were analyzed using hamster to infer the ancestral state. Random intergenic (dotted line) is the same as Additional file 1, Fig. S3C–D. **E**–**F** Similar to **A**–**B** except that 4655 dog and fox genes were analyzed using bear to infer the ancestral state. Random intergenic (dotted line) is the same as Additional file 1, Fig. S3E–F

Thus far we have examined the current static picture of GC-content surrounding the TSS. To determine the nucleotide substitution dynamics, we used comparative phylogenetic analysis. We aligned DNA sequences 2500 bp upstream and downstream of the TSS of homologous genes in humans and chimpanzee to identify substitution events. To infer the ancestral state, we compared these alignments to the gorilla homolog. Strikingly, we observed that GC-content surrounding the TSS of protein-coding genes is decaying in both humans and chimpanzee (Fig. 4A, B). This can be seen even when examining transition substitutions (Additional file 1, Fig. S2A–B). Note that fluctuations in the total number transition substitutions around the TSS (e.g., A to G and T to C) are due to changes in nucleotide content. When we computed the nucleotide substitution rates by dividing each substitution event over the total mutable nucleotides (e.g., [G to A]/[G]), many of these fluctuations in substitution rates, especially A to G and T to C, are dampened near the TSS (Additional file 1, Fig. S2G–H), suggesting that the overall loss in GCs was partially due to the presence of fewer mutable A/T nucleotides surrounding the TSS. When random intergenic sequences were analyzed, GC-content was slightly increasing (Additional file 1, Fig. S3A, B, the averages of these were plotted in Fig. 4A, B, dashed lines). We next performed permutation tests between the change of GC at TSSs and intergenic sites that were either GC-matched or not, and observed that the GC-loss at TSSs was significantly greater than at the random intergenic sites but not the GC-matched sites (Additional file 1, Fig. S3G). This is consistent with the idea that the GC-peak is experiencing decay at roughly the same rate as intergenic regions with equivalently high GC-content.

To validate these results, we then examined substitutions surrounding the TSSs of mouse and rat genes, using hamster to infer the ancestral state. Again, we observed a decrease in GC-content surrounding the TSS (Fig. 4C, D). This can also be seen in the total number of transition substitutions (Additional file 1, Fig. S2C–D). When we computed the nucleotide substitution rates, some of the fluctuations damped, but they were still somewhat pronounced in rat (Additional file 1, Fig. S2I–J). Again this loss was much higher than at random intergenic sites but not statistically different from GC-matched intergenic sites (Additional file 1, Fig. S3G). Interestingly, we observe that GC-content is rapidly growing in the genome of rodents, especially in rats (Additional file 1, Fig. S3C–D).

Although GC-content seemed to decrease at the TSS, it is known to increase near recombination sites due to gBGC [29]. To confirm this, we examined documented recombination hotspots in the mouse genome [52], using rat and hamster genomes to infer the substitution dynamics. Indeed, we observed an increase in GC-content in these regions (Additional file 1, Fig. S4A) that was above the rate for GC-matched intergenic regions (Additional file 1, Fig. S4B, average is replotted as a dashed line in both S4A and S4B).

From these analyses, we concluded that GC-content at the TSS of protein-coding genes is not at equilibrium, but in decay in primates and rodents. This decay rate is similar to the decay seen in intergenic regions that have the same GC-content (Additional file 1, Fig. S3G), this despite the fact that TSSs likely experience fewer CpG deamination events [26]. Using estimates of divergence between the various organisms, and the number of substitutions, we estimate that this decay in GC-content is on the order of 1% every 10 million years. This implies that the GC-peak built up to some higher point in our evolutionary past, but has since then been in decline.

### De novo mutations at human protein-coding gene TSSs show a loss of GC-content

**Fig. 5:**
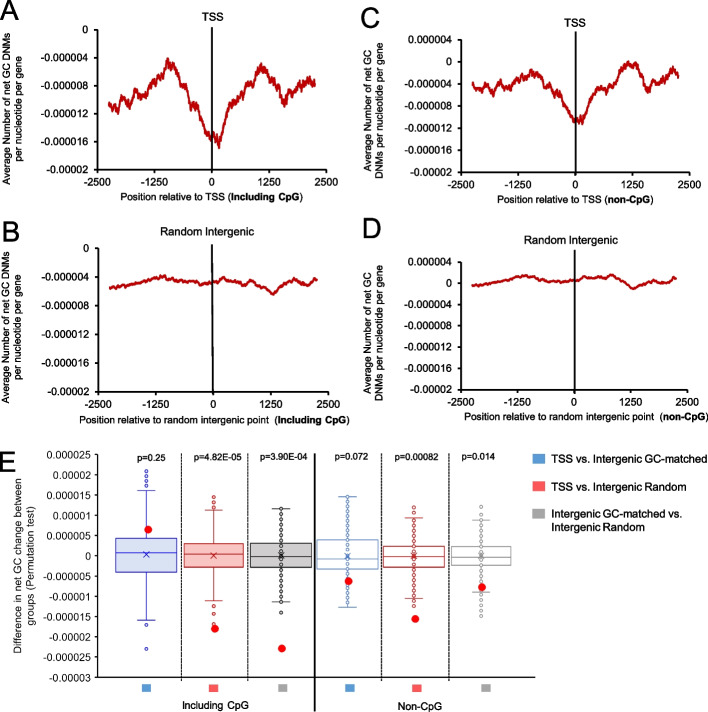
Changes in GC-content due to de novo mutations surrounding human TSSs according to parent-offspring trio analyses. **A** De novo mutations surrounding the TSS of human protein-coding genes were compiled from Jónsson et al. [53] and divided by the number of genes analyzed (*y-axis*) using a sliding window of 100 bp and plotted along genomic regions surrounding the TSS (*x-axis*). **B** Similar to **A** except that random intergenic sequences were analyzed. **C**–**D** Similar to **A**–**B** except that C to T and G to A mutations in CpGs were omitted. **E** Permutation test results comparing net change of GC substitutions around the 0 point for each treatment group in **A**–**D**. Distribution of differences from 1000 randomized permutations are displayed in box and whisker plots. Actual differences are plotted with red dots. *p* values are indicated

Previously, we examined nucleotide substitutions between related species. To further validate the decay of GC-content at the TSS, we mapped human germline de novo mutations from 1548 parents/offspring trios [53] to regions surrounding protein-coding gene TSSs. In agreement with our substitution analysis, we observed that regions around the TSS had a greater number of mutations that reduced GC-content than gained GC-content (Fig. 5A). This difference was significant when compared to random intergenic regions (Fig. 5B, E solid red bar) but not random GC-matched regions (Fig. 5E solid blue bar), suggesting that the peak is undergoing decay at the same rate as regions of the genome that are high in GC-content but not under any selective constraint. This difference could also be seen when total transition mutations were analyzed (Additional file 1, Fig. S5A for TSS, Fig. S5C for intergenic). We could also see an enhanced number of mutations away from GC at regions further away from the TSS which appeared as shoulders in Fig. 5A (nucleotides less than −1250 and greater than 1250). We next computed the nucleotide mutation rates and we observed that transition mutation rates away from G and C were slightly depressed around the TSS (Additional file 1, Fig. S5B) compared to the rates in intergenic regions (Additional file 1, Fig. S5D). These rates rose at either end, likely explaining the shoulders in Fig. 5A.

It was previously observed that CpG decay due to spontaneous deamination was suppressed around TSSs [26], likely due to CpG hypomethylation, and this could explain the suppression of G to A and C to T that we observe around the TSSs (Additional file 1, Fig. S5B). To test this, we compiled all non-CpG mutations (TSS shown in Fig. 5C, and in Additional file 1, Fig. S5E, F, intergenic controls shown in Fig. 5C, and Additional file 1, Fig. S5G, H) and found that the excess of mutations away from GC around the TSS was still apparent, however, the shoulders seen in Fig. 5A were less apparent (Fig. 5C). This loss in GC for non-CpG mutations was also significant when compared to random intergenic regions (compare Fig. 5C to D, compare Additional file 1, Fig. S5E to S5G, also see stats in Fig. 5E empty red bar), but not significant when compared to a GC-matched set of intergenic regions (Fig. 5E empty blue bar). Overall, our analysis of de novo mutations confirms that human TSSs are losing GC-content due to mutational decay to a lower equilibrium level.

### Canids are experiencing a gain of GCs surrounding protein-coding gene TSSs

We next examined the GC-dynamics of canids. As described above, PRDM9 became a pseudogene in this clade, and as a result recombination occurs at TSSs in these organisms. We compared sequences from the dog and fox genomes, using bear (which is not a canid) to infer the ancestral state. In marked contrast to what we observed in primates and rodents, the regions surrounding canid protein-coding gene TSSs are gaining GC-content (Fig. 4E, F). Again, this trend is also apparent in transition substitutions, both in their total numbers (Additional file 1, Fig. S2E–F) and in their rates (Additional file 1, Fig. S2K–L), although there appeared to be a depression of A to G and T to C mutations right around the TSS. The suppression of these mutations may explain the overall dip in GC-gain right in the vicinity of canid TSSs, especially in the fox genome. This dip could be due to negative selection eliminating certain mutations from promoters and exons, and this is somewhat supported by decreases in the other two transition mutations (G to A and C to T, Additional file 1, Fig. S2E–F, K–L). However, it is also possible that these alterations are due to biased mutation/repair events in the vicinity of the TSS. Despite all these trends, the overall rate of GC-increase in these regions was higher than the genome average, as compiled by measuring the change in GC-content in random intergenic regions (Additional file 1, Fig. S3E–F, the averages were plotted in Fig. 4E, F, dashed lines). When the changes were compared to random GC-matched intergenic regions, they were still higher, with the possible exception of the regions right around fox TSSs (Additional file 1, Fig. S3G, black and green bars). These changes were statistically significant (Additional file 1, Fig. S3G).

These data further show that recombination leads to a local increase in GC and that the presence or absence of a functional *PRDM9* gene dictates whether regions surrounding protein-coding gene TSSs are gaining or losing GC-content in mammals.

### Biases due to recombination affect codon usage in the open reading frame

Previously we observed a GC-peak at the 5′ end of human mRNAs even if we restrict ourselves to the ORF (Additional file 1, Fig. S1). Thus, we examined whether changes in GC-content, caused by the presence or lack of recombination, extend to the ORF and affect codon choices. Both adaptive and non-adaptive theories, although not mutually exclusive, have been used to explain synonymous codon usage in humans [51]. The first theory proposes that synonymous codon usage co-adapted with the abundance of tRNAs to optimize the efficiency of translation [54]. The second theory proposes that synonymous codon usage depends on the large-scale fluctuations of GC-content along chromosomes [55].

**Fig. 6:**
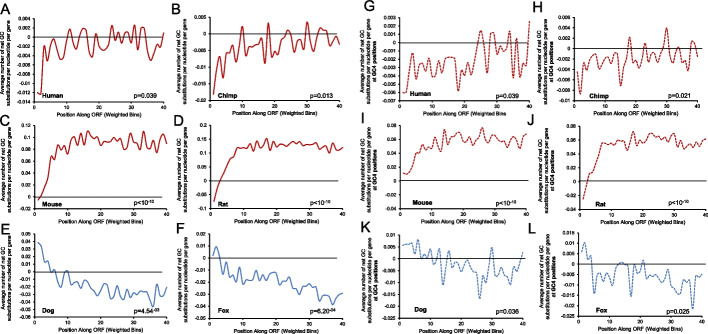
Net change of GC substitutions along the ORF. Average number of the net change of GC substitutions along the ORF normalized to 40 bins were plotted for human (**A**), chimpanzee (**B**), mouse (**C**), rat (**D**), dog (**E**), and fox (**F**), with a sliding window of 100 bp. Average number of the net change of GC substitutions along the ORF at 4-fold degeneracy positions (GC4) normalized to 40 bins were plotted for human (**G**), chimpanzee (**H**), mouse (**I**), rat (**J**), dog (**K**), and fox (**L**), with a sliding window of 100 bp. Only genes with the ORF starting in exon 1 and the start codon (ATG) were included in the analysis. *p* values are from permutation tests of changes in GC-content between the beginning (bin 1) of the ORF compared to the middle (bin 20) of the ORF (see “Methods”)

We performed the same trio-species nucleotide substitution analysis for the entire length of the ORF of all analyzed genes normalized to 40 bins. We observed that human, chimp, mouse, and rat (species that have PRDM9 and perform recombination at hotspots) have a significantly higher relative loss of GC-content at the beginning in comparison to downstream portions of the ORF (Fig. 6A–D). Interestingly, rodents are gaining GC-content throughout the majority of their ORF, which is in line with our previous data suggesting rodents (especially rats) are gaining GC-content throughout their genome (Additional file 1, Fig. S3C–D). In species that do not have PRDM9 and perform recombination at the TSS (dog and fox), there is a significant net gain in GC-content at the beginning of the ORF which diminishes as one travels downstream (Fig. 6E, F). We then compared changes in GC-content from ORF confined to either the first exon (and thus close to the TSS) or the fourth exon. We limited our analysis to genes whose start codons occur in the first exon, which accounts for approximately 40% of all human protein-coding genes [48]. We observed that human, chimp, mouse, and rat all have a significantly higher loss of GC-content in coding sequence from the first exon compared to the fourth exon, whereas dog and fox show the opposite trend (Additional file 1, Fig. S6A, Table S1). These results suggest that biases dues to the presence or lack of recombination near the TSS affect changes in GC-content that extend into the beginning of the coding regions of genes.

Next, we examined whether mutational bias affects synonymous codon choice. In particular, we monitored changes in GC-content at 4-fold degeneracy sites of codons (GC4) along the ORF. In agreement with our other findings, we observed that primates and rodents are losing more GCs at GC4 sites at the beginning of the ORF compared to downstream regions (Fig. 6G–J), whereas canids show the opposite trend (Fig. 6K, L). These observations are all significantly different with the exception of dog, although the same trend is still present (Additional file 1, Fig. S6B, Table S1). Taken together, we show that changes in GC-content in the coding regions and synonymous codon positions are affected by the presence or lack of recombination at the TSS.

## Discussion

Although there is a tendency in the life sciences to ascribe all features to natural selection, it is clear, but not widely appreciated, that non-adaptive processes also contribute to evolution [56–59]. In this manuscript, we present evidence that the GC-content of protein-coding gene TSSs is influenced, in part, by local rates of recombination. It is possible that local GC-content around the TSS is also subject to selection as high GC-content can promote transcription and mRNA nuclear export. However, it appears that selective forces act in conjunction with (or in some cases in opposition to) non-adaptive processes to shape these GC-peaks. Importantly, our data shows that in the absence of recombination, the GC-peak is decaying at a rate that is similar to DNA regions that have similar levels of GC-content. Since most intergenic regions are non-functional, this decay of GC-content at the TSS is consistent with neutral evolution. Thus, selection alone is not strong enough to maintain the GC-peak surrounding the TSS in mammalian protein-coding genes.

**Fig. 7:**
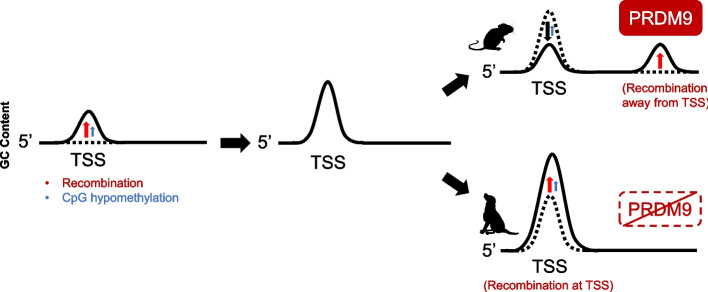
Model for the evolution of GC-peaks at protein-coding gene TSSs. Recombination shapes GC-content at the TSS. The GC-peak in the TSS was built up due to recombination and likely to some extent CpG hypomethylation (repression of CpG decay) near promoters. Since then, PRDM9 is directing recombination away from the TSS, leading to a decrease in GC-content at the TSS for primates and rodents due to mutational decay (black arrow). In organisms that lost PRDM9, recombination occurs at the TSS, promoting further increase in GC-content in canids

Our data suggests a model outlined in Fig. 7. GC-peaks were likely built up due to evolutionary forces acting on the TSS regions of protein-coding genes in the ancestors of humans and likely all amniotes. It is unlikely that these GC-peaks first arose due to positive selection, which was then relaxed leading to their present day decay. This would require that GC-content play a vital role in the expression of genes in the common ancestor, but not so in organisms such as apes and rodents where these GC-peaks are decaying. Instead, we believe that these peaks were likely caused, and partially maintained, by two main forces. First, CpGs that surround the TSS are hypomethylated and thus partially protected from the loss of cytosines by spontaneous deamination. Indeed, it has been observed that CpG decay is repressed around TSSs [26]. This form of biased mutation can also explain the prevalence of CpG islands in GC-rich regions without invoking selection [60]. Secondly, we believe that in most amniotic lineages, recombination occurred at the TSS of protein-coding genes during distinct time intervals, which were interspersed by other periods where recombination was directed away from TSSs. These distinct periods were likely due to the rapid evolution of PRDM9 [40]. Interestingly, snakes, which have a functional PRDM9, perform recombination at both TSSs and PRDM9-binding sites [61]. It is believed that there is a tug-of-war competition between TSSs-associated factors and PRDM9 for recruiting downstream factors in the recombination pathway, and that in snakes the strength of these two complexes is somewhat balanced [62]. The rapid evolution of PRDM9 may allow different variants, each differing in their relative strength in this tug-of-war competition, to appear in succession within a given lineage. This would cause periodic increases in GC-content at TSSs, in a punctuated manner, followed by intervals of slow decay. Thus, the current alleles of PRDM9 that are present in human, chimp, mouse, and rat are very effective at directing recombination away from the TSS to PRDM9-dictated hotspots, and thus these organisms are currently experiencing a decay in their GC-peaks to a lower equilibrium state. Canids, which do not have PRDM9, perform recombination at the TSS, and this further drives up GC-content in these regions to a higher equilibrium state.

This model is supported by findings in a recent preprint, which documents the equilibrium state of GC-content in TSS regions from numerous mammals [63]. In this study, the authors compute the equilibrium GC-content state at TSSs by measuring nucleotide substitution rates in closely related trio species (similar to the analysis we performed to generate Fig. 4 and Supplementary Fig. 2). They find that for many mammals, the equilibrium GC-content state is consistent with some level of recombination at protein-coding TSSs, even in those species that have PRDM9. Unsurprisingly, species with the highest inferred rates of TSS-associated recombination are in the canids clade. Species with the lowest inferred rates of TSS-associated recombination are humans, mice, and cetaceans. This suggests that most mammals experience some boost in GC-content surrounding the TSS due to a certain degree of recombination there despite the fact that these organisms contain functional versions of PRDM9 [63].

At this stage, we are uncertain about the evolutionary history of certain features of the TSS-associated GC-peak. The GC-peak appears to be widespread throughout amniotes, although it is not as prominent in anole lizards. Other branches of the vertebrate tree, such as boney fishes, lack a clear GC-peak. Interestingly, it has been recently reported that GC-content is not elevated near recombination hotspots in many teleost fish, suggesting that their genomes do not undergo strong gBGC [64]. Indeed, the European sea bass has a truncated form of PRDM9 and performs recombination at TSSs [64], and lacks a GC-peak at protein-coding TSSs (Marie Raynaud, personal communication). The lack of GC-peaks at TSSs is most likely a particularity of teleost fish as other branches of the vertebrate tree that are more distantly related to amniotes, such as lampreys, have clear GC-peaks at their TSSs that are likely due to local and historic trends in recombination, although this must be verified. The association between TSSs, recombination, and GC-content is found in even more distantly related organisms. For example, certain plants have a similar 5′ to 3′ gradient of GC-content in their protein-coding genes [65, 66] and these have recently been linked to recombination and gBGC [67]. Thus, TSS-associated recombination and its impact on GC-content may be a widespread phenomenon throughout eukaryotes. It is likely that there is a deep connection between TSSs and recombination, perhaps because transcription initiation near the TSS can cause DNA double stranded breaks [68–70], which can be used to initiate recombination. The association of TSSs and recombination may be more emphasized in certain lineages than others, explaining its patchwork presence throughout the eukaryotic tree.

Finally, our work suggests that certain features of genes, which are recognized by cellular machinery, may be largely shaped by non-adaptive processes. Thus, the high GC-content that is found at the start of protein-coding genes, which is used to promote efficient nuclear export of certain mRNAs, appears to be shaped by historic patterns of recombination. It is generally assumed that such “functional” genomic features are at the very least maintained by purging selection. Our new results suggest that some of these features, which appear to specify functional parts of the genome, could be present regardless of selective forces. Instead, parts of the cellular machinery, in this case the mRNA export machinery, seemed to have evolved to recognize these patterns that are largely generated by non-adaptive process.

It is likely that non-adaptive forces may create other genomic features that are exploited by the cellular machinery for functional ends [1, 59]. These non-adaptive evolutionary forces may help to generate very strong signals in genomes that experience weak selection regimes.

## Conclusion

The dynamics of the GC-peak that surrounds the TSS of protein-coding genes in amniotes is largely shaped by historic patterns of recombination. Since decay of GC-content towards the mutation rate equilibrium is the default state for non-functional DNA, the observed decrease in GC-content at TSSs in apes and rodents indicates that the GC-peak is not being maintained by selection on most protein-coding genes in those species. Our results indicate that non-adaptive evolutionary forces help to generate certain features associated with functional genomic regions. Some of these features can then be recognized by cellular machinery, such as mRNA export factors, for functional ends.

## Methods

### Sequence data and annotation

Sequences of animal genomes were accessed and downloaded from the Ensembl database (www.ensembl.org). The genome assembly versions are listed in Table S2. Annotations for protein-coding genes were retrieved using the University of Santa Cruz (UCSC) Genome Browser annotation track database based on the corresponding genome assemblies. For human and mouse, annotations for the TSS were obtained from the FANTOM5 project, which uses Cap Analysis of Gene Expression (CAGE) sequencing [71]. The best TSS from CAGE-seq was defined as the transcript with the highest tags per million score. For all other organisms, annotations for the best TSS were determined by the most commonly used transcript and start site obtained from Ensembl (http://www.ensembl.org/info/data/ftp/index.html/). The exon/intron boundaries are obtained from the corresponding best TSS. Sequences surrounding the TSS were retrieved using the BEDTools suite (GitHub: get_homologous_sequences.py). Similarly, intergenic genome regions were retrieved by randomly generating genome coordinates outside of protein-coding regions (GitHub: generate_random_coordinates.py). Intergenic GC-matched sequences were obtained by selecting from the list of random intergenic sequences, a new set of sequences that have the same distribution of GC-content as the dataset that it is being matched to (GitHub: gc_match.py). Annotations for the frequency of recombination rate of protein-coding genes were obtained from the HapMap genetic map and divided into the top 10% and bottom 10% of recombination rates (RRID: SCR_002846) [51, 72]. Annotations for mouse recombination hotspots were obtained from previously published datasets [52]. Sequences for the open reading frame were obtained using codes from the GitHub repository by Graham E Larue (https://github.com/glarue/cdseq). Genes with their ORF starting in exon 1 were selected by filtering for ORF sequences with start coordinates between the TSS and first exon/intron boundary (ORF_start_exon1.py).

### Binning strategy and GC-content analysis

To assess the GC-content of sequences, we divided the sequence track into different numbers of equally sized bins: 20 bins for mRNA sequences, 41 bins for sequences surrounding the TSS and exon/intron boundary, and 40 bins for ORF sequences. For sequences surrounding the TSS and exon/intron boundary, we calculated the size of the first exon, and obtained size-matched sequences of the corresponding first intron, and two times the size-matched sequences of the corresponding upstream intergenic regions. The 41 bins consist of 20 upstream, one at the TSS, and 20 downstream (10 exonic and 10 intronic). We subsequently calculated the GC percent for each bin (GitHub: gc_content_binning.py). Whenever multiple isoforms of the same gene are present, the GC-content for that gene is the weighted average of all isoforms (e.g., for a gene with 4 isoforms, each isoform contributes to 25% of the GC-content for that gene).

### Sequence alignment

Homologous sequences were aligned between primate trios (human, chimpanzee, gorilla), rodent trios (mouse, rat, hamster), and carnivora trios (dog, fox, bear). For sequences surrounding the TSS in protein-coding genes, homologous genes were obtained from Ensembl biomart. For sequences in random intergenic regions and recombination hotspots, triple homologous search was performed using standalone Basic Local Alignment Search Tool (BLAST) from the National Center for Biotechnology Information (NCBI) (GitHub: get_blast_sequences.py). Triple sequence alignment was performed using the Needleman Alignment algorithm (GitHub: needleman_alignment.py). A 60% alignment cutoff was applied to all sequence alignments. The total number of trio gene/sequence alignments obtained after the cutoffs used for mapping is in Table S3.

### Nucleotide substitution mapping

Gorilla was used as the reference sequence for substitutions in human and chimpanzee, hamster as the reference sequence for substitutions in mouse and rat, and bear used as the reference sequence for dog and fox. Nucleotide substitutions were identified based on the above triple alignments. Transition substitutions, or net change in GC substitutions per gene or sequence analyzed were mapped with a 100-nucleotide sliding window surrounding the TSS, random intergenic site, or recombination hotspot. For rates of substitutions, the number of single substitutions was divided by the total number of that nucleotide of interest per position (e.g., number of A to G substitutions for position 1, divided by the total number of A nucleotides in all genes at position 1). Subsequently a 100-nucleotide sliding window is applied (GitHub: find_substitutions.py). For the GC4 positions in the ORF, we used only ORF sequences that begin with the start codon “ATG” with sequence lengths divisible by 3. GC substitutions for ORF GC4 positions were extract based substitutions that fall at a position where the sequence is a 3rd synonymous codon position (GitHub: find_substitutions_orf_gc4.py). For total substitutions found in exon 1 or exon 4 of the ORF, we used only ORF sequences that contain 4 or more exons (GitHub: count_substitutions_per_exon.py).

### De novo mutation mapping

Human germline de novo mutations from 1548 parents/offspring trios were obtained from [53] and mapped to 5 kb surrounding the best TSS (defined by FANTOM5 CAGE-seq, see “Sequence data and annotation” section) of protein-coding genes. DNMs were separated into whether or not they occur in a CpG dinucleotide. Rate of DNMs was calculated by dividing the number of DNMs by the number of total mutable nucleotides, similarly to the above nucleotide substitution mapping (GitHub: DNM.ipynb).

### Statistical analysis

To statistically test whether the GC-content at one locus of a sequence is significantly different from the GC-content at another locus, the Wilcoxon signed-rank test is performed between the distributions of the 2 loci (GitHub: wilcoxontest.py).

To statistically test whether the number of net change in GC substitutions or DNMs at relative nucleotide position 0 is significantly different between two datasets (e.g., between the TSS and random intergenic position), a permutation test is performed. In this test, net change in GC substitutions or DNMs from datasets A and B are randomly shuffled into two groups 1000 times, and the differences between the two random groups from the 1000 randomizations are used to generate a normalized curve. The actual observed delta between datasets A and group B is plotted onto the normalized curve, and the *p* value is the area under the curve separated by the line x = observed delta (GitHub: permutation_test.py). Permutation tests have been described previously (Wilcox, 2022).

### Supplementary Information

Additional file 1. This file contains all supplementary figures (Figs. S1 through S6).

Additional file 2. This file contains all supplementary tables (Tables S1 through S3).

Additional file 3. This file contains all supplementary tables (Tables S1 through S3).

## Data Availability

The code presented in the paper has been implemented and is available for public access on GitHub: https://github.com/tinaqiu221/GC_evolution [77]. All datasets can be obtained from the Zenodo repository: https://zenodo.org/records/10694966 [78]. The code and datasets are distributed under the MIT open-source license.
