# Supplementary material for: The GC-content at the 5′ ends of human protein-coding genes is undergoing mutational decay
Source: Genome Biol. 2024 Aug 13;25:219. doi: 10.1186/s13059-024-03364-x (PMC11323403; doi:10.1186/s13059-024-03364-x)
Supplement: Supplementary file 1 — Additional file 1. This file contains all supplementary figures (Figs. S1 through S6). [file 13059_2024_3364_MOESM1_ESM.docx]

**
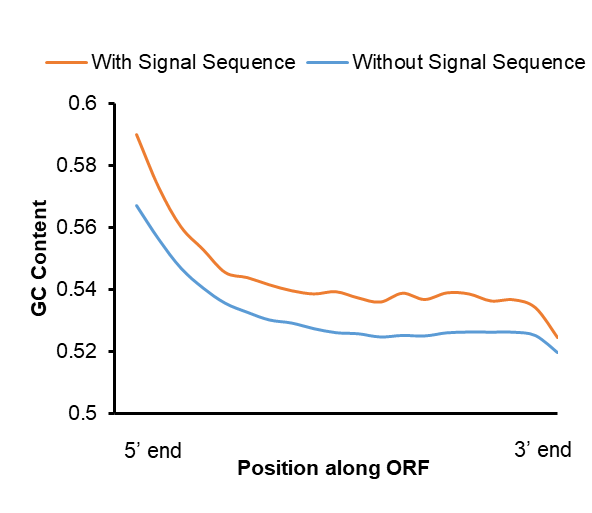
**

**Figure S1. GC content in the open reading frame.**

The average GC-content for all human protein coding genes (*y-axis*) was plotted against the normalized open reading frame length (from 5’ end to 3’ end in 20 bins; *x-axis*), further divided into genes that contain a signal sequence coding region (N=5754) and those without (N=17627).

**
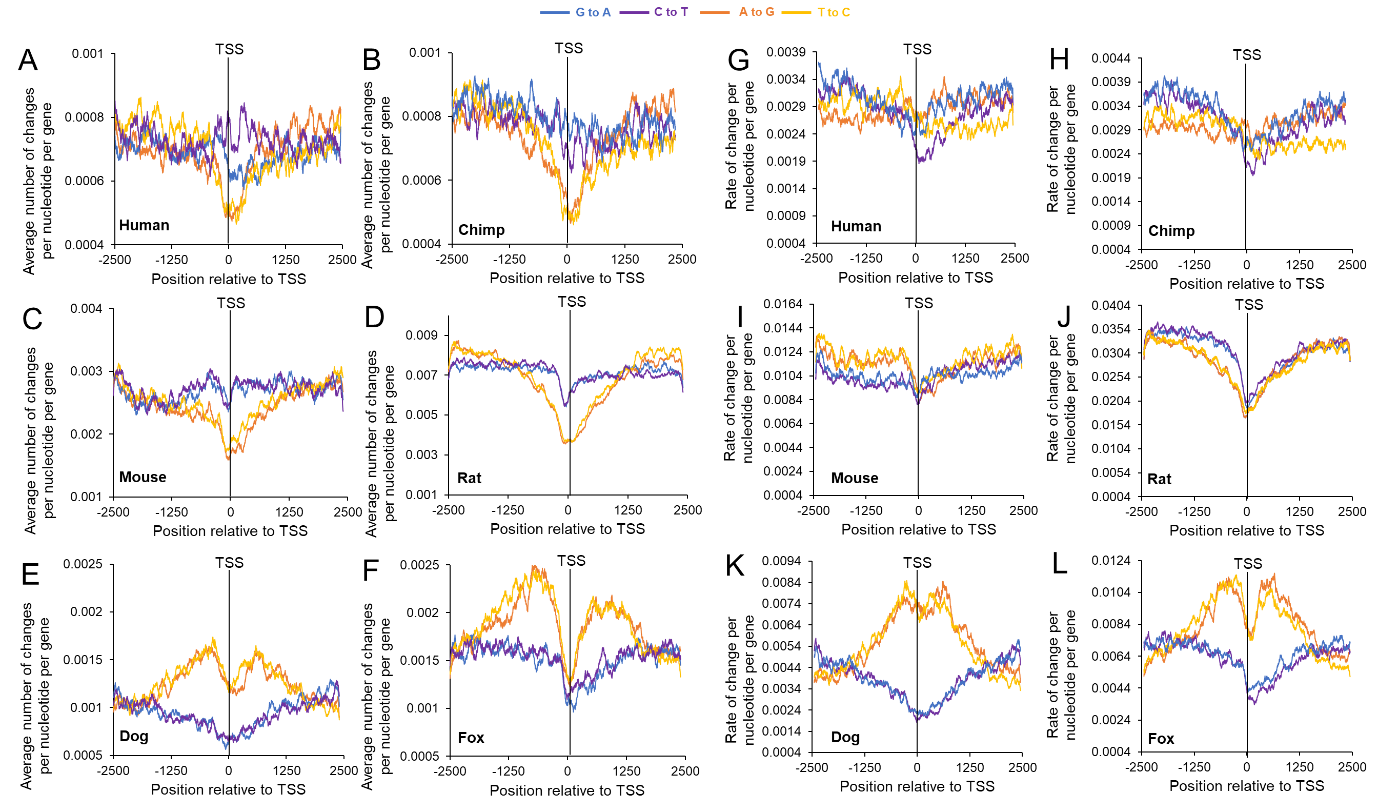
**

**Figure S2. Transition substitution numbers and rates surrounding TSSs from various mammalian genomes according to comparative phylogenetic analyses.**

A-F) Average number of A to G (orange), T to C (yellow), G to A (blue) and C to T (purple) substitutions in human (A), chimpanzee (B), mouse (C), rat (D), dox (E), and fox (F) divided by the number of genes analyzed (*y-axis*) using a sliding window of 100 bp and plotted along genomic regions surrounding the TSS (*x-axis*).

G-L) Rates of each substitution (number of nucleotide substitutions/total mutable nucleotides, e.g., [G to A]/[G]) from A-F were plotted around the TSS for the species indicated.


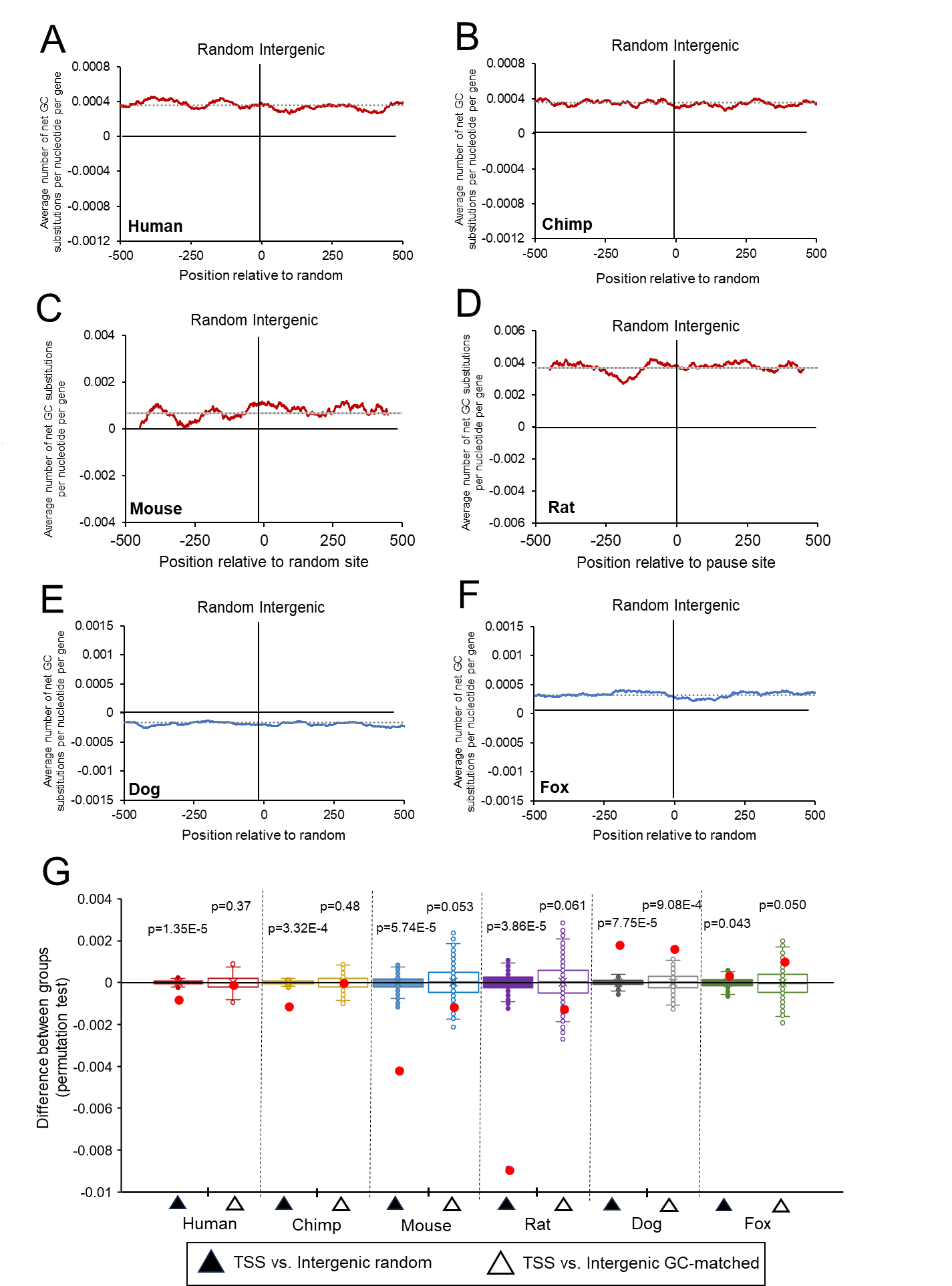


**Figure S3. Changes in GC-content surrounding intergenic regions from various mammalian genomes according to comparative phylogenetic analyses.**

A-F) The total change in Gs and Cs in human (A), chimpanzee (B), mouse (C), rat (D), dox (E), and fox (F) were compiled and divided by the number of genes analyzed (*y-axis*) using a sliding window of 100 bp and plotted along genomic regions surrounding random intergenic points (*x-axis*). Total number of intergenic regions is equal to the number of analyzed genes for each species in Figure 4A-F. The overall average change in GC-content for each plot (dotted line) were plotted – these are also plotted in Figure 4A-F.

G) Permutation test results comparing net change of GC substitutions around the 0 point for each treatment group in (A-F). Distribution of differences from 1000 randomized permutations are displayed in box and whisker plots. Actual differences are plotted with red dots. P-values are indicated. Intergenic GC-matched plots are not shown.

**
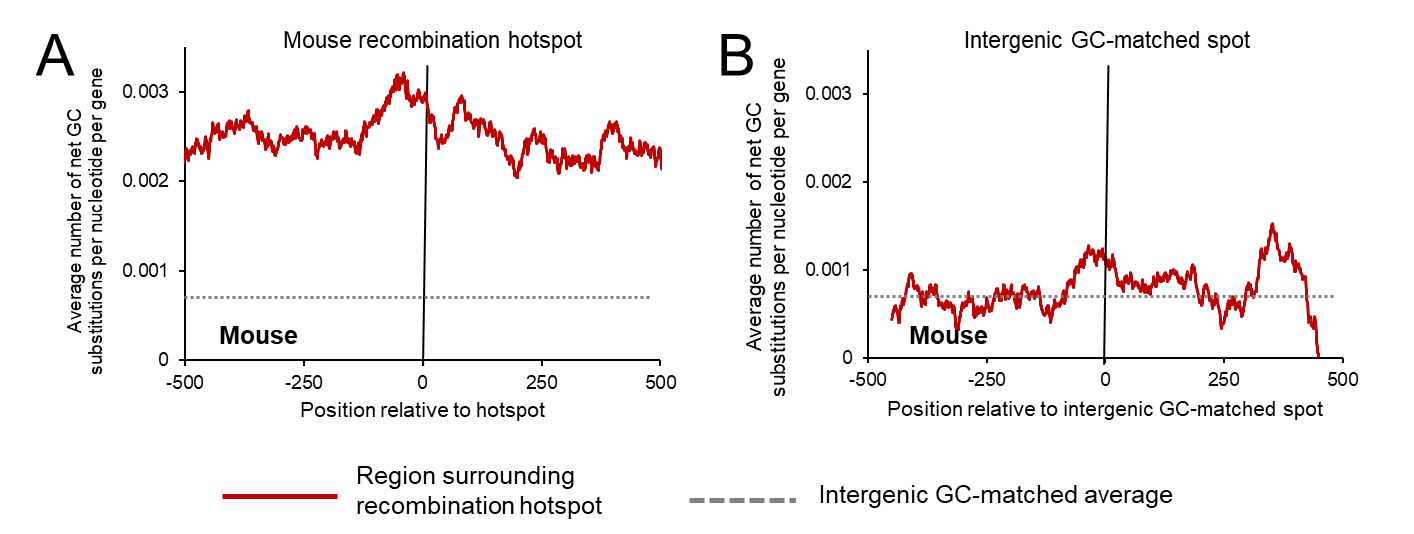
**

**Figure S4. Changes in GC-content surrounding mouse recombination hotspots according to comparative phylogenetic analysis.**

A) The total change in Gs and Cs in mouse from mouse-rat-hamster trio analysis, were compiled surrounding recombination hotspots (N=2668). Change in G and C divided by the number of regions analyzed was plotted (*y-axis*) over a sliding window of 100 bp along genomic regions surrounding recombination hotspots (*x-axis*). The overall average change in GC-content in mouse at a random intergenic spot (dotted line) was plotted.

B) Similar to (A) except plotted along intergenic regions that are GC-matched to the GC content of house recombination hotpots (*x-axis*) (N=4543).

**
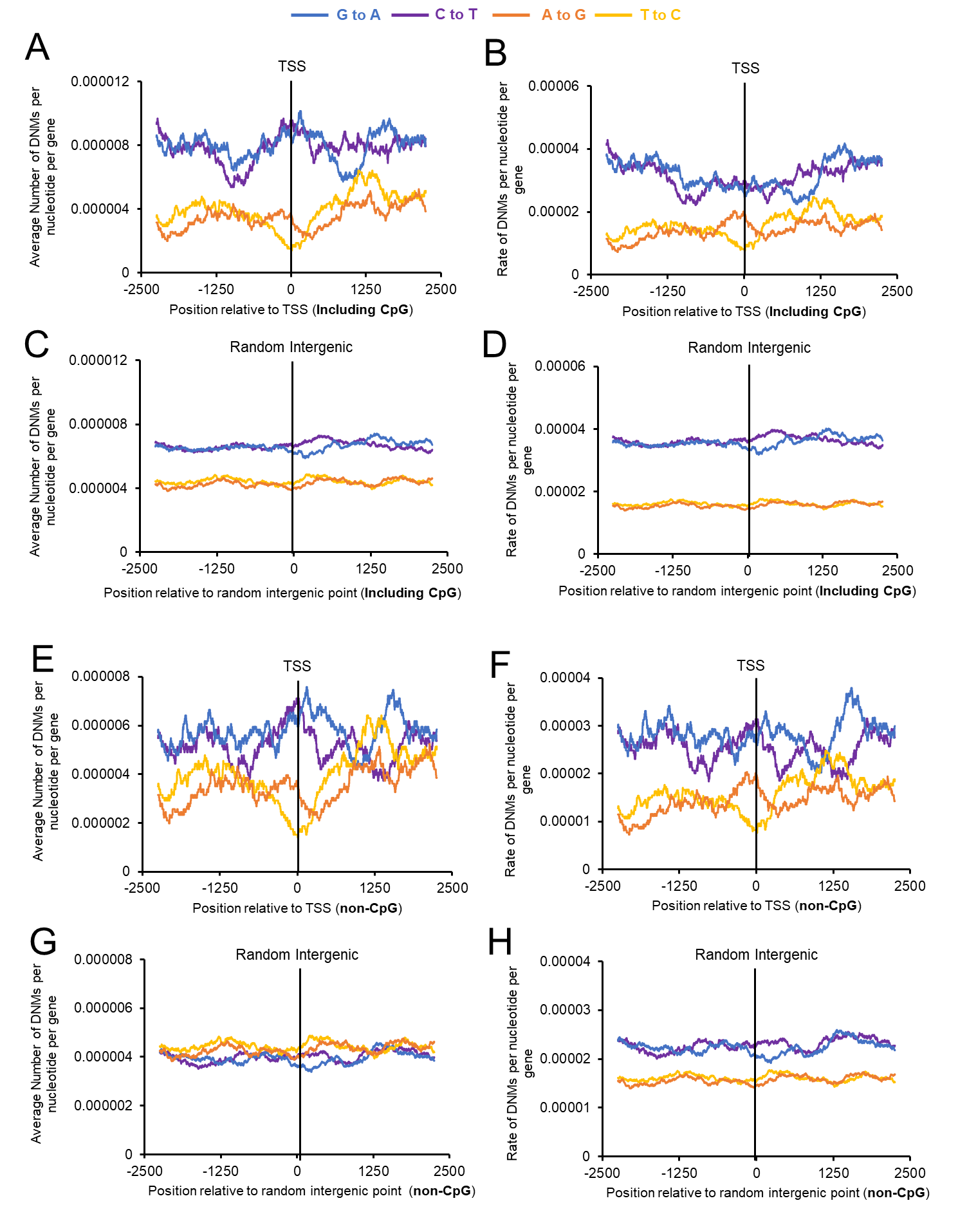
**

**Figure S5. Transition *de novo* mutations, numbers and rates, surrounding human TSSs according to parent-offspring trio analyses.**

A) Average number of A to G (orange), T to C (yellow), G to A (blue) and C to T (purple) human *de novo* mutations divided by the number of genes analyzed (*y-axis*) using a sliding window of 100 bp and plotted along genomic regions surrounding the TSS (*x-axis*).

B) Rates of each *de novo* mutation (number of nucleotide substitutions/total mutable nucleotides, e.g., [G to A]/[G]) from (A).

C-D) Similar to (A-B) except plotted around random intergenic region (*x-axis*).

E-H) Similar to (A-D) except that C to T and G to A mutations in CpGs were omitted.

**
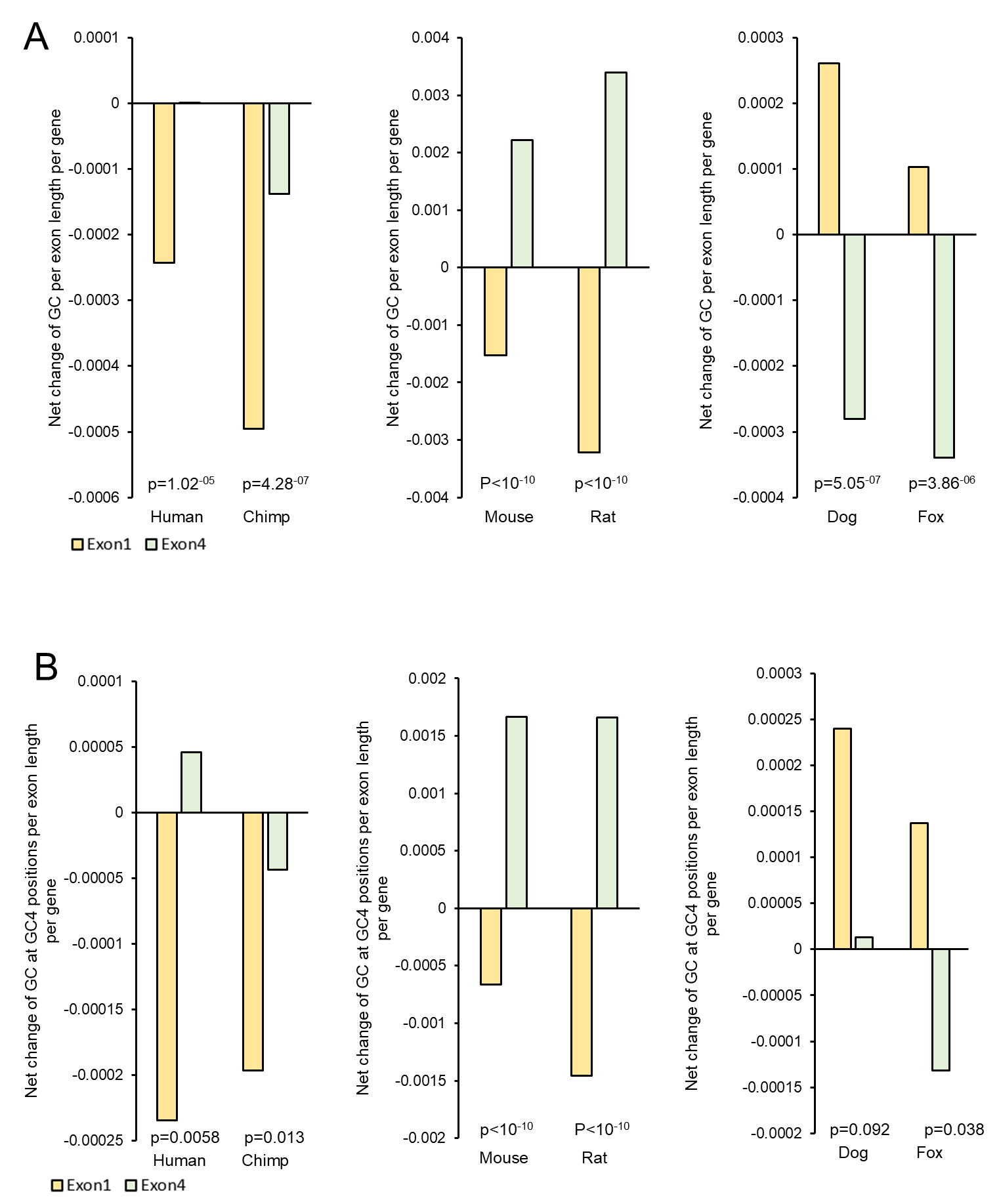
**

**Figure S6. Change in GC content in exon 1 and exon 4 of protein coding genes in different organisms according to comparative phylogenetic analyses.**

A) The total change in Gs and Cs in exon 1 and exon 4 of genes, divided by the lengths of the corresponding exons and the total number of genes analyzed (Table 3). Only genes with the ORF starting in exon 1, and genes that contain at least 4 exons, were included in the analysis.

B) Similar to (A) except only analyzing 4-fold degenerate codon positions (GC4) in exon 1 and exon 4 (Table 3). P-values are from Wilcoxon signed-rank tests of changes in GC content between exon 1 and exon 4 of protein coding genes in different organisms (see methods, Table 1).
