# Supplementary material for: The GC-content at the 5′ ends of human protein-coding genes is undergoing mutational decay
Source: Genome Biol. 2024 Aug 13;25:219. doi: 10.1186/s13059-024-03364-x (PMC11323403; doi:10.1186/s13059-024-03364-x)
Supplement: Supplementary file 2 — Additional file 2. This file contains all supplementary tables (Tables S1 through S3). [file 13059_2024_3364_MOESM2_ESM.docx]

**TABLES**

**Table S1.** **Wilcoxon signed-rank test statistics of changes in GC content between exon 1 and exon 4 of protein coding genes in different organisms according to comparative phylogenetic analyses.**

|  | **Human** | **Chimp** | **Mouse** | **Rat** | **Dog** | **Fox** |
| --- | --- | --- | --- | --- | --- | --- |
| Observed delta (Net change of GC between exon1 and exon 4) | -0.00024 | -0.00036 | -0.00374 | -0.00662 | 0.000541 | 0.000443 |
| p-value | 1.02E-05 | 4.28E-07 | 2.10E-46 | 7.04E-136 | 5.05E-07 | 3.86E-06 |
| Observed delta (Net change of GC between exon 1 and exon 4 at GC4 positions) | -0.00028 | -0.00015 | -0.00233 | -0.00312 | 0.000227 | 0.000269 |
| p-value (GC4) | 0.005823 | 0.012921 | 4.65E-28 | 1.04E-54 | 0.092441 | 0.037624 |

**Table S2. Genome releases and assembly versions.**

| **Organism** | **Genome assembly version** |  |
| --- | --- | --- |
| Human (*Homo sapien*) | GRCh38 |  |
| Chimpanzee (*Pan troglodytes*) | Pran_trio3.0 |  |
| Gorilla (*Gorilla gorilla*) | gorGor4 |  |
| Mouse (*Mus musculus*) | GRCm39 |  |
| Rat (*Rattus norvegicus*) | mRatBN7.2 |  |
| Hamster (*Mesocricetus auratus*) | MesAur1.0 |  |
| Dog (*Canis lupus familiaris*) | ROS_Cfam_1.0 |  |
| Fox (*Vulpes vulpes*) | VulVul2.2 |  |
| Bear (*Ursus americanus*) | ASM334442v1 |  |
| Chicken (*Gallus gallus*) | bGalGal1.mat.broiler.GRCg7b |  |
| Crocodile (*Crocodylus porosus*) | CroPor_comp1 |  |
| Turtle (*Pelodiscus sinensis*) | PelSin_1.0 |  |
| Tortoise (*Chelonoidis abingdonii*) | ASM359739v1 |  |
| Lizard (*Podarcis muralis*) | PodMur_1.0 |  |
| Frog (*Xenopus tropicalis*) | UCB_Xtro_10.0 |  |
| Toad (*Leptobrachium leishanense*) | ASM966780v1 |  |
| Coelacanth (*Latimeria chalumnae*) | LatCha1 |  |
| Zebrafish (*Danio rerio*) | GRCz11 |  |
| Shark (*Callorhinchus milii*) | Callorhinchus_milii-6.1.3 |  |
| Lamprey (*Petromyzon_marinus*) | Pmarinus_7.0 |  |
| Sponge (*Amphimedon queenslandica*) | v1.1 |  |

**Table S3. Number of trio gene/sequence alignments used for mapping.**

| **Genome region** | **Organism** | **Number of trio genes/sequences** |
| --- | --- | --- |
| TSS | Human/Chimp | 9248 |
|  | Mouse/Rat | 4840 |
|  | Dog/Fox | 4655 |
| Intergenic random | Human/Chimp | 20209 |
|  | Mouse/Rat | 6142 |
|  | Dog/Fox | 8000 |
| Intergenic GC-matched to TSS | Human/Chimp | 400 |
|  | Mouse/Rat | 509 |
|  | Dog/Fox | 470 |
| Recombination hotspots | Mouse/Rat | 2668 |
| Intergenic GC-matched to recombination hotspots | Mouse/Rat | 4543 |
| ORF | Human/Chimp | 17690 |
|  | Mouse/Rat | 18890 |
|  | Dog/Fox | 15074 |
| ORF (GC4) | Human/Chimp | 14394 |
|  | Mouse/Rat | 8932 |
|  | Dog/Fox | 5876 |
| ORF (Exon 1 and Exon 4) | Human | 5433 |
|  | Chimp | 9240 |
|  | Mouse | 5531 |
|  | Rat | 7113 |
|  | Dog | 6624 |
|  | Fox | 7558 |
| ORF (Exon 1 and Exon 4, GC4) | Human | 4400 |
|  | Chimp | 7648 |
|  | Mouse | 2784 |
|  | Rat | 3912 |
|  | Dog | 2189 |
|  | Fox | 2992 |
