# Supplementary material for: The GC-content at the 5′ ends of human protein-coding genes is undergoing mutational decay
Source: Genome Biol. 2024 Aug 13;25:219. doi: 10.1186/s13059-024-03364-x (PMC11323403; doi:10.1186/s13059-024-03364-x)
Supplement: Supplementary file 3 — Additional file 3. This file contains all supplementary tables (Tables S1 through S3). [file 13059_2024_3364_MOESM3_ESM.docx]

***Genome Biology* review history**

**Reviewer 1** (same reviewer as Reviewer 1 on Review Commons)

The revised manuscript has improved in clarity. I am happy with the manuscript as is. Thanks for following up on the reviewer's comments.

I am now able to follow the arguments as to the observation that CG-content being higher at TSS of species that lack PRDM9 (which allows recombination at TSS) and lower at species with PRDM9 (which prevent recombination at TSS). This observation leads to the hypothesis that it is recombination the force behind high GC at TSS. The argument that the observed GC-peak in humans and rodents were caused by past recombination at TSS that were permitted by ancient variants of PRDM9 seems to me possible but quite speculative.
